# Gut microbiota responses to two-dimensional materials: insights from *in vivo* models and *in vitro* antibacterial studies

**DOI:** 10.3389/ftox.2026.1865240

**Published:** 2026-09-07

**Authors:** Wen Xu, Guotao Peng

**Affiliations:** College of Environmental Science and Engineering, Key Laboratory of Yangtze River Water Environment, Shanghai Institute of Pollution Control and Ecological Security, State Key Laboratory of Water Pollution Control and Green Resource Recycling, Tongji University, Shanghai, China

**Keywords:** 2D materials, gut microbiota, microbial communities, nano-microbiota interactions, physicochemical properties

## Abstract

Two-dimensional (2D) materials are increasingly applied in environmental and biomedical fields, raising concerns about their interactions with the gut microbiota following oral exposure. In this review, we summarize current understanding of the effects of 2D materials, ranging from graphene-based materials (GBMs) to emerging 2D materials such as transition metal dichalcogenides (TMDs) and transition metal carbides and nitrides (MXenes), on the gut microbiota and the associated biological outcomes. We first summarize *in vivo* studies, mainly using mouse and zebrafish models and focusing on GBMs, demonstrating that 2D materials exposure can induce gut microbiota dysbiosis, often in association with local and systemic host responses. To provide mechanistic insights, we further examine how key physicochemical properties, particularly nanosheet dimensions, surface chemistry, and material transformation, determine the interactions between 2D materials and bacterial cells, drawing from the extensive body of *in vitro* antibacterial research. Finally, we highlight future research directions beyond the need for more efforts on emerging 2D materials, including linking physicochemical properties of 2D materials to microbial responses *in vivo* and analyzing the temporal response patterns of the gut microbiota. We also emphasize the development of community-relevant *in vitro* models and the need to dissect the potential biotransformation of 2D materials by the gut microbiota. A deeper understanding of the interactions between 2D materials and the gut microbiota will not only advance the safety assessment of these materials but also foster the development of safer-by-design 2D materials.

## 1 Introduction

Two-dimensional (2D) materials have attracted extensive attention because of their unique layered structures and exceptional physicochemical properties, such as large specific surface areas, tunable lateral dimensions, and adjustable surface chemistries (Lyu et al., 2022; Guan et al., 2026). These features have promoted their use in food-related technologies, including food packaging and contaminant detection (Hashim et al., 2022), as well as in biomedical platforms for delivery, imaging, and therapy (Zhang et al., 2026), and in environmental remediation through adsorption or catalytic pollutant removal (Garg et al., 2021). This widespread exploration and application, however, raises concerns about their potential environmental and health impacts, as they can be released and enter the human body via ingestion, inhalation, or dermal contact.

While the safety assessment of graphene-based materials (GBMs) has been intensively investigated, particularly regarding their potential human and environmental impacts (Fadeel et al., 2018), the toxicological understanding of other emerging 2D materials, including hexagonal boron nitride (hBN), transition metal dichalcogenides (TMDs), transition metal carbides and nitrides (MXenes), and black phosphorus (BP), remains comparatively limited (Peng and Fadeel, 2022; Lin et al., 2024). Current studies on these materials have primarily focused on cellular responses or *in vitro* antibacterial activity (Mei et al., 2020; Wu et al., 2022), whereas their gastrointestinal effects *in vivo* remain largely unexplored. This evidence gap is particularly concerning given that these materials differ substantially from GBMs in their elemental composition, surface chemistry, and environmental transformation behavior, which may influence their biological interactions and toxicity profiles. Moreover, emerging evidence suggests that certain transition metal-containing 2D materials, such as MXenes and TMDs, can release metal ions under physiological or environmental conditions (Tang et al., 2020; Peng et al., 2023a), potentially introducing additional toxicity pathways beyond those associated with the parent nanosheets.

Under an oral exposure scenario, the intestine represents a major site of contact for 2D materials. Even during inhalation, some ingestion can occur, also leading to intestinal exposure (Pietroiusti et al., 2016). The intestine is not only an exposure site, but also a complex biological interface composed of the epithelial barrier, mucus layer, immune components, and commensal microbiota (Yan et al., 2024). Much of the current evidence relevant to intestinal exposure has described 2D material toxicity through selected endpoints, including antibacterial effects (Sahoo and De, 2022), inflammatory responses (Jia et al., 2019), and microbiota disturbance (Liu et al., 2021). These studies have provided important baseline information, but they often rely on simplified bacterial models or terminal *in vivo* measurements, making it difficult to determine how the gut microbiota, mucus layer, epithelial barrier, and mucosal immunity change over the course of exposure. This temporal dimension is important because these intestinal barriers may not respond in parallel, and the primary mechanism underlying toxicity may shift over time.

Within the intestinal microenvironment, the diverse community of commensal gut microbiota plays a critical role in sensing external stimuli and modulating the host immune system and metabolism (Nicholson et al., 2012; Lynch and Pedersen, 2016). Consequently, dysbiosis of the gut microbiota is closely associated with the onset of various diseases, including inflammatory bowel disease, arthritis, and diabetes (Kamada et al., 2013). In the context of 2D material exposure, the gut microbiota may act not only as a direct target of material interactions, but also as a mediator linking material exposure to host responses. For instance, our recent study demonstrated that exposure to graphene oxide can reshape the gut microbiota in zebrafish, triggering type 2 immune responses via the activation of the aryl hydrocarbon receptor (AhR) by the microbial metabolite butyrate (Peng et al., 2023b). This finding underscores the complex pathway from material exposure to host response in the gut. As we have recently highlighted, a critical challenge lies in uncovering the specific correlations between the physicochemical properties of environmental pollutants and the resulting gut microbial dysbiosis, as well as the molecular mechanisms that link these microbial changes to adverse host outcomes (Gai et al., 2026).

**FIGURE 1:**
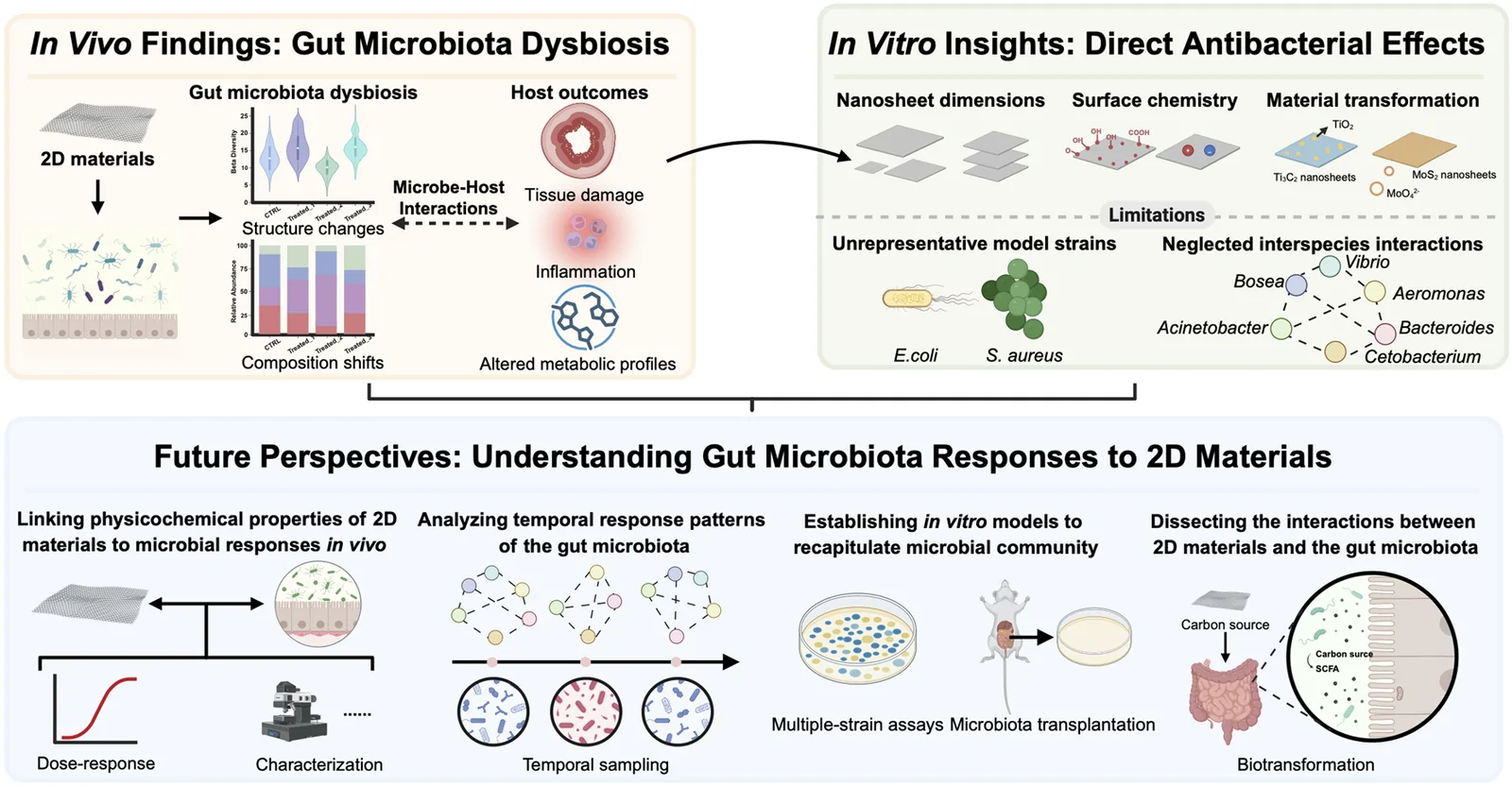
Schematic summary of current findings and future perspectives on gut microbiota responses to 2D materials. Created with BioRender.com.

Here, we focus on summarizing the current understanding of the effects of 2D materials on the gut microbiota and the associated biological consequences, with representative studies organized in Table 1. Because *in vitro* antibacterial studies provide useful mechanistic clues on direct interactions between materials and bacteria, we also discuss how key physicochemical properties influence bacterial responses. Finally, we identify key gaps that need to be addressed to better connect material properties, microbiota dynamics, and biological outcomes. These include extending investigations beyond GBMs to emerging 2D material classes, improving *in vivo* structure-activity analyses, incorporating temporal designs, developing community-relevant *in vitro* models, and considering the transformation of 2D materials driven by the microbiota (Figure 1).

**TABLE 1:** Summary of representative *in vivo* and *in vitro* studies on gut microbiota and bacterial responses to 2D materials.

Materials	Physicochemical properties	Experimental model	Exposure methods and doses	Toxicity responses	Ref.
*In vivo*					
Graphene (GR)	Characterized by scanning electron microscope (SEM) and transmission electron microscopy (TEM), but lateral size and layer number were not specified	6-week-old male ICR mice	Oral gavage for 4 weeks; 1, 10, or 100 μg/day graphene	Altered gut microbial diversity and community composition, with a stronger effect at 1 μg/day than at higher doses	(Xie et al., 2016)
GR	Characterized by atomic force microscopy (AFM); small few-layer graphene (S-FLG: lateral size mainly 20–70 nm, thickness≈1.05 nm, mainly 3 layers); large few-layer graphene (L-FLG: lateral size mainly 300–700 nm, thickness≈1.4 nm, mainly 4 layers)	Adult zebrafish (*Danio rerio*)	Waterborne exposure to^14^C-labeled S-FLG or L-FLG; depuration and tissue distribution assays: 75 μg/L for 48 h, with or without natural organic matter, followed by depuration in clean water; cellular uptake assay: 250 μg/L for 48 h; gut microbiota response assay: 5 mg/L for 2 weeks	L-FLG was mainly retained in the digestive tract and efficiently eliminated during depuration, whereas S-FLG was detected in intestinal epithelial cells, blood, and liver. S-FLG caused stronger alterations in intestinal microbial diversity and community structure than L-FLG.	(Lu et al., 2017)
GR; graphene oxide (GO); reduced graphene oxide (rGO)	Characterized by AFM for lateral size and thickness, and by Raman for I_D_/I_G_; GR (lateral size≈0.508 μm, thickness≈1.58 nm, I_D_/I_G_ = 0.90); GO (lateral size≈0.297 μm, thickness≈1.76 nm, I_D_/I_G_ = 0.86); rGO (lateral size≈41.5 nm, thickness≈13 nm, I_D_/I_G_ = 0.92)	Adult zebrafish (*Danio rerio*)	Dietary exposure for 21 days; 1 μg/day	GR significantly decreased gut microbial diversity; GR, GO and rGO shifted gut microbial community structure	(Zheng et al., 2019)
GO	Characterized by TEM for morphology, dynamic light scattering (DLS) for lateral size, AFM for thickness; average lateral size: 321.74 nm; thickness: 0–1.2 nm	Adult zebrafish (*Danio rerio*)	Waterborne exposure for 25 days; 0.05, 0.5 and 5 mg/L	GO exposure disturbed intestinal microbial diversity and composition, damaged tissues, and induced intestinal inflammatory responses	(Jia et al., 2019)
GO	TEM-measured lateral size: 0.1–15 μm; AFM-measured thickness: 1–2 nm	WT and *ahr2* ^+/−^ adult zebrafish (*Danio rerio*); conventional and germ-free (GF) zebrafish larvae	Adult zebrafish: continuous exposure to GO at 50 or 500 μg/L for 7 days; larvae: GO plus butyrate exposure for 24 h to examine AhR activation and immune responses	GO altered adult zebrafish gut microbiota in an AhR genotype dependent manner; GO combined with butyrate activated intestinal AhR signaling and recruited ILC-like immune cells in GF larvae	(Peng et al., 2023b)
GO	Characterized by TEM, DLS and AFM; ∼99% single-layer ratio; Hydrodynamic diameter: 236 ± 5 nm in DI water and 766 ± 179 nm in simulated intestinal fluid; Thickness: <2 nm in water and nearly 5 nm in simulated intestinal fluid	Pregnant ICR mice	Oral gavage during gestational days 7–16; 2, 10 and 40 mg/kg/day; Fecal microbiome was analyzed on GD19 in the control and 40 mg/kg groups	GO exposure altered maternal gut microbiome, with reduced microbial diversity in the high-dose group, and was accompanied by placenta barrier dysfunction and adverse pregnancy outcomes	(Liu et al., 2021)
Nano-MoS_2_; micro-MoS_2_	Reported by supplier: nano-MoS_2_ (0.02–1 μm, thickness 1–10 nm); micro-MoS_2_ (∼1.5 μm)	Male C57BL/6 mice	Dietary exposure for 90 days; food premixed with nano-MoS_2_ or micro-MoS_2_ at 15 or 150 mg MoS_2_/kg food	Altered gut microbiota and gastrointestinal metabolic profiles; micro-MoS_2_ effects were mainly linked to microbiota alterations, whereas nano-MoS_2_ also involved direct intestinal toxicity	(Wu et al., 2019)
Ti_3_C_2_T_x_	Characterized by TEM, zeta potential, and hydrodynamic size; irregular flakes with diameter ≈100 nm; zeta potential ≈−18 mV; hydrated sheet diameter ≈1.8 μm	*Daphnia magna*	0.01 or 1 mg/L Ti_3_C_2_T_x_. Growth and gene expression assays: 1-day-old neonates exposed for 7 days. Gut microbiota assay: 14-day-old individuals exposed for 1 day	Inhibited growth and development, suppressed growth-related genes, and induced gut microbiome dysbiosis	(Xiang et al., 2026)
*In vitro*					
GO	Monolayer GO sheets characterized by AFM; average lateral area decreased with sonication time: GO-0, 0.753 μm^2^; GO-10, 0.127 μm^2^; GO-30, 0.065 μm^2^; GO-50, 0.035 μm^2^; GO-120, 0.013 μm^2^; GO-240, 0.010 μm^2^	*Escherichia coli* (*E. coli*) K12	*E. coli* was incubated with GO suspensions in deionized water at 40 μg/mL for 2 h	GO-0 showed the strongest antibacterial activity, whereas GO-240 showed the weakest	(Liu et al., 2012)
GO	Average sheet thickness characterized by AFM: 1.4 nm; SEM-measured average sheet areas decreased with sonication time: 0.65, 0.29, 0.10 and 0.01 μm^2^	*E. coli* K12	GO-coated surface assay: GO-coated filters prepared by filtering 2 mL of 200 μg/mL GO suspension; *E. coli* contacted the surface for 3 h. Suspension assay: *E. coli* exposed to 200 μg/mL suspended GO for 3 h	Size dependent antibacterial behavior varied with exposure conditions: 0.65 μm^2^ GO showed stronger apparent inhibition in suspension, whereas 0.01 μm^2^ GO showed stronger bactericidal activity on coated surfaces	(Perreault et al., 2015)
GO	Size-varied GO suspensions prepared by different sonication conditions; DLS-measured hydrodynamic sizes: GO-1, 1,295 nm; GO-2, 2,015 nm; GO-3, 3,074 nm; GO-4, 4,544 nm	*Streptococcus mutans* (*S. mutans*)	*S. mutans* was exposed to GO at 25 μg/mL for 30 min	GO-2 showed the strongest antibacterial activity, suggesting a parabolic size-activity relationship	(Yu et al., 2020)
Graphite (Gt), graphite oxide (GtO), GO, and rGO	DLS-measured nominal effective diameters: Gt, 5.25 μm; GtO, 4.42 μm; GO, 0.56 μm; rGO, 2.93 μm; SEM-measured nominal particle sizes: Gt, 6.87 ± 3.12 μm; GtO, 6.28 ± 2.50 μm; GO, 0.31 ± 0.20 μm; rGO, 2.75 ± 1.18 μm	*E. coli*	*E. coli* was incubated with Gt, GtO, GO or rGO dispersions at 40 μg/mL for 2 h	Antibacterial activity followed GO > rGO > Gt > GtO	(Liu et al., 2011)
GO	Fully exfoliated, flat GO sheets (∼1 nm thickness, AFM); one-, two-, and three-layer LB films, with increased deposition supported by higher UV-Vis absorbance and XPS C 1s intensity	*E. coli* K12 MG1655	GO-LB films were inoculated with *E. coli* suspension at 10^7^ CFU/mL and incubated for 2 h; antibacterial activity was assessed using an *in situ* live/dead assay	Bacterial inactivation increased with deposited layer number; the three-layer film achieved 89% inactivation, compared with 13% for bare PET.	(Mangadlao et al., 2015)
GO coatings on Ti	Prepared by cathodal electrophoretic deposition at 40, 80, or 120 V (GO-40, GO-80, and GO-120); coating thickness, characterized by cross-sectional SEM: ∼25, 90, and 136 μm	*Staphylococcus aureus* (*S. aureus*), ATCC 25923; *E. coli*, ATCC 25922	Coated Ti surfaces were inoculated with 60 μL bacterial suspension (10^7^ CFU/mL) and incubated at 37 °C for 24 h	GO coatings markedly reduced bacterial survival; increasing layer number was accompanied by higher reactive oxygen species (ROS) levels and stronger antibacterial activity	(Qiu et al., 2017)
Partially reduced graphene oxide (prGO) nanosheets	Prepared from AACVD-derived prGO films by liquid-phase exfoliation for 48–216 h; lowest reported thickness: 6.18 nm at 120 h (AFM)	Clinical burn isolates: *Pseudomonas aeruginosa* (*P. aeruginosa*), *E. coli*, *Acinetobacter baumannii (A. baumannii)*, and *S. aureus*	Agar well-diffusion assay; 50 μL prGO dispersion per well; bacterial inoculum: 0.5 McFarland (≈1.5 × 10^8^ CFU/mL); 37 °C for 24 h	All four isolates were inhibited; 120 h preparation produced the largest inhibition zones	(Baqi et al., 2026)
MoS_2_	Size-separated MoS_2_ nanosheets prepared by gradient centrifugation; DLS-measured sizes: 409, 307 and 215 nm	*E. coli*	*E. coli* suspension was incubated with size-separated MoS_2_ nanosheets for 3 h	Antibacterial activity increased as MoS_2_ size decreased from 409 to 215 nm	(Zhao et al., 2021)
Chemically exfoliated MoS_2_ sheets (ce-MoS_2_) and restacked ce-MoS_2_ aggregates	AFM showed monolayer ce-MoS_2_ sheets with thickness ≈1 nm and most sheets >200 nm. SEM showed restacked ce-MoS_2_ aggregates with layered morphology	*E. coli* DH5α	Aggregation assay: *E. coli* DH5α was exposed to freshly dispersed ce-MoS_2_ or restacked/aggregated ce-MoS_2_ at 20 μg/mL for 2–6 h	Well-dispersed ce-MoS_2_ showed stronger antibacterial activity, whereas restacked/aggregated ce-MoS_2_ showed reduced activity	(Yang et al., 2014)
Mixed-charge functionalized 2D-MoS_2_ nanosheets	Surface functionalization was achieved using different ratios of positively and negatively charged thiol ligands: 10P, 8P2N, 6P4N, 5P5N and 10N. Zeta potential confirmed tunable surface charge: 10P, +23 mV; 8P2N, +11.9 mV; 6P4N, –0.04 mV; 5P5N, –24.1 mV; 10N, –42.8 mV	Gram-positive methicillin-resistant *Staphylococcus aureus* (MRSA) and Gram-negative *P. aeruginosa*	Broth dilution antibacterial assay using mixed-charge functionalized 2D-MoS_2_	Mixed-charge functionalization produced Gram-selective antibacterial activity. 6P4N preferentially inhibited MRSA, while 8P2N preferentially inhibited *P. aeruginosa*	(Sahoo and De, 2022)
Vertically aligned MoS_2_ nanosheet coatings on Ti	Vertically aligned, edge exposed MoS_2_ nanosheets; coating thickness: ∼330 and 790 nm for MoS-1 and MoS-2, respectively (cross-sectional SEM). Ion release in physiological saline was quantified by ICP-AES.	*E. coli*, ATCC 25922 and *S. aureus*, ATCC 25923	Bacteria were exposed to aqueous molybdate (MoO_4_^2-^, 60 ppm) for 24 h; viability was assessed by alamarBlue™.	Molybdate inhibited *S. aureus* but showed no obvious inhibitory effect on *E. coli*	(Tang et al., 2020)
Few-layered MoS_2_ nanosheets; sodium molybdate (Na_2_MoO_4_) as a soluble Mo control	Lateral size: 136.00 ± 79.28 nm (TEM)	*E. coli *RK 4353 wild-type (WT)	MoS_2_: 25, 50, 100, and 200 μg/mL; Na_2_MoO_4_: 32, 64, 128, and 256 μg/mL at equivalent Mo concentrations; with or without NaNO_3_ (10 mM). Bacterial growth was monitored at 37 °C for 800 min	MoS_2_ inhibited *E. coli* growth in a dose-dependent manner, and the presence of NaNO_3_ further enhanced this effect. Na_2_MoO_4_ did not inhibit bacterial growth under the same conditions	(Peng et al., 2023a)
Ti_3_C_2_T_x_	SEM-characterized average lateral sizes: 0.09, 0.35, 0.57 and 4.40 μm	*E. coli* and *Bacillus subtilis* (*B. subtilis*)	Bacterial suspensions were treated with 100 μg/mL Ti_3_C_2_T_x_ nanosheets for 3 or 8 h in the dark	Antibacterial activity increased as Ti_3_C_2_T_x_ lateral size decreased; 0.09 μm nanosheets caused stronger membrane damage and DNA release	(Arabi Shamsabadi et al., 2018)
Pristine 2D Ti_3_C_2_ MXene flakes and poly-L-lysine-modified Ti_3_C_2_ flakes (Ti_3_C_2_/PLL)	Zeta potential showed PLL adsorption reversed the surface charge from negative to positive: pristine Ti_3_C_2_, −5.6 mV; Ti_3_C_2_/PLL, +44.9 mV	*E. coli* MG 1655	Antibacterial assay: *E. coli* MG1655 was exposed to pristine Ti_3_C_2_ or Ti_3_C_2_/PLL flakes for 6 h at 25 °C; material concentrations were tested by two-fold dilution	Pristine Ti_3_C_2_ showed little antibacterial activity, whereas Ti_3_C_2_/PLL reduced viable *E. coli* cells by approximately two orders of magnitude at 200 mg/L	(Rozmysłowska-Wojciechowska et al., 2020)
Ti_3_C_2_T_x_ MXene-coated polyvinylidene fluoride (PVDF) membranes	Prepared by vacuum-assisted filtration; 1.2 μm Ti_3_C_2_T_x_ coating on PVDF. Air aging for over 30 days induced surface oxidation with TiO_2_/C formation	*E. coli* and *B. subtilis*	Filtration assay: 50 μL bacterial suspension (10^4^ CFU/mL) diluted to 10 mL and filtered through the membranes, followed by 24 h incubation at 35 °C	Fresh membranes inhibited *E. coli* and *B. subtilis* growth by 67% and 73%, respectively. After air aging, inhibition exceeded 99% for both strains	(Rasool et al., 2017)
Graphdiyne (GDY) and graphdiyne oxide (GDYO)	AFM-measured thickness: GDY, 4–9 nm; GDYO, 4–11 nm. DLS-measured hydrodynamic size: GDY, ≈410 nm; GDYO, ≈370 nm. XPS showed increased oxygen-containing groups in GDYO: C-O increased from 11.21 to 21.70 mol%, and C=O from 2.79 to 12.50 mol%	*E. coli* and *S. aureus*	Main antibacterial assays used 3.0 mg/mL GDY/GDYO and 10^5^ CFU/mL bacteria	Surface oxidation converted weakly active GDY into antibacterial GDYO.	(Zhang et al., 2019)

## 2 *In vivo* models: gut microbiota dysbiosis and host responses

Existing evidence demonstrating the effects of 2D materials on gut microbiota alterations and associated host physiological responses has mainly focused on GBMs across different animal models (Table 1). Early efforts showed that oral administration of GR to mice for 4 weeks resulted in significant shifts in gut microbiota composition and diversity (Xie et al., 2016). Interestingly, the strongest perturbation of the microbial community was observed at the lowest exposure level (1 μg/d) compared to 10 and 100 μg/d groups, suggesting that the influence of GR on gut microbiota does not follow a simple dose-dependent pattern. This phenomenon was attributed to aggregation of GR at higher concentrations, which may limit its effective interaction with microorganisms.

Physicochemical characteristics of GBMs influence microbial responses in the intestine. For instance, GR nanosheets with different lateral sizes exhibited distinct intestinal behaviors (Lu et al., 2017). With comparable thickness (3–4 layers), S-FLG (lateral size mainly 20–70 nm) showed a greater capacity to cross the intestinal barrier, whereas L-FLG (lateral size mainly 300–700 nm) was mainly retained in the gut lumen and eliminated during depuration. Consistent with these differences, S-FLG exposure induced more pronounced alterations in microbial diversity and community structure than L-FLG. Distinct microbial responses have also been observed among GBMs with different oxidation states. Dietary exposure to GR (I_D_/I_G_ = 0.90), GO (I_D_/I_G_ = 0.86), and rGO (I_D_/I_G_ = 0.92) induced different dysbiosis patterns in the gut microbiota of zebrafish (Zheng et al., 2019). GR exposure significantly reduced microbial alpha diversity, whereas GO and rGO did not produce statistically significant changes in diversity. Nevertheless, all three materials induced shifts in microbial community structure.

Moving beyond describing microbiota alterations, growing efforts have been made to uncover the potential links between gut microbiota dysbiosis and host physiological responses upon exposure (Zickgraf et al., 2023). For example, chronic exposure (25 days) to GO (lateral size 321.74 nm, thickness 0–1.2 nm) markedly altered the gut microbial community in zebrafish and was accompanied by intestinal inflammation (Jia et al., 2019). Specifically, GO exposure increased the relative abundance of *Proteobacteria*, a phylum widely considered a hallmark of microbial dysbiosis. In contrast, taxa such as *Bacteroidetes* and *Fusobacteria*, which are involved in nutrient metabolism and intestinal homeostasis, were reduced. At the genus level, potentially pathogenic bacteria including *Vibrio* and *Pseudomonas* became more abundant, while beneficial bacteria such as *Bacillus* and *Akkermansia* were reduced. These microbial alterations were suggested to contribute to tissue damage and intestinal inflammatory responses, evident by the upregulation of proinflammatory genes. Also using zebrafish as model organisms (Peng et al., 2023b), we exposed adult zebrafish to GO (lateral size 0.1–15 μm, thickness 1–2 nm) for 7 days and found that GO could change the composition of the gut microbiota and trigger a type 2 immune response. More strikingly, these responses were dependent on the AhR genotype of the fish. By using germ-free zebrafish larvae, we further confirmed the role of microbial metabolite butyrate in this process, showing that GO endowed with butyrate as a “corona” activated AhR signaling in the intestinal epithelium and induced the recruitment of immune cells, particularly innate lymphoid cell (ILC)-like cells. In addition, microbiota dysbiosis has been reported together with systemic physiological effects in mammalian models (Liu et al., 2021). Oral exposure to GO during pregnancy in mice caused pronounced placenta barrier dysfunction and adverse pregnancy outcomes, which were suggested to be associated with alterations in the maternal gut microbiome, including decreased microbial diversity and significant shifts in community composition.

Regarding other emerging 2D materials, one study reported that mice exposed to MoS_2_ through long-term dietary administration exhibited noticeable changes in the gut microbial community and altered metabolic profiles of the gastrointestinal tract. The authors further analyzed the associations and found that the intestinal metabolic changes observed in the micro-MoS_2_ (lateral size ∼1.5 μm) group were largely related to microbiota alterations. In contrast, the effects of nano-MoS_2_ (lateral size 0.02–1 μm) appeared to involve not only microbiota perturbation but also direct intestinal toxicity of the material (Wu et al., 2019). Very recently, a related observation has also been reported for MXene materials (Xiang et al., 2026). In *Daphnia magna* exposed to Ti_3_C_2_T_x_, the intestinal microbial community structure was significantly altered, and the exposed organisms simultaneously showed growth and developmental inhibition, including reduced body length and decreased molting frequency. Changes in bacterial taxa involved in nutrient metabolism were also observed. These results indicate that disruption of the gut microbiota occurred together with impaired growth and development in the exposed organisms.

Collectively, although the effects of GBMs on gut microbiota have been studied to a certain extent, variations in physicochemical properties and model organisms leave the establishment of structure-activity relationships largely unknown. In addition, most *in vivo* studies characterize dispersion-related properties before exposure, such as hydrodynamic size and zeta potential, whereas the aggregation or dispersion state of 2D materials within the gastrointestinal environment remains poorly defined. Moreover, whether gut microbiota dysbiosis acts as a driver or a consequence of host outcomes remains unclear, since current *in vivo* studies rarely separate direct material-microbe interactions from host-mediated microbial changes. Germ-free animals and microbiota manipulation models are therefore needed to clarify this causal relationship. Furthermore, more efforts are needed on other emerging 2D materials, as findings obtained from GBMs may not apply to all layered materials, particularly those containing transition metals that may be bioavailable and thus have more complicated biological fates (Peng et al., 2022).

## 3 Mechanistic insights from *in vitro* antibacterial studies

When *in vivo* observations reveal significant alterations, including decreases in particular bacterial taxa, direct interactions between 2D materials and bacteria represent one possible explanation. For instance, in a study of chronic GO exposure in zebrafish, *in vitro* assays were conducted using bacterial strains isolated from the zebrafish intestine, showing that GO could directly affect the survival of these intestinal bacteria (Jia et al., 2019). This finding supports the possibility of direct GO-bacterium interactions, but does not by itself explain the community-wide changes observed *in vivo*. Current understanding of the antibacterial activity of 2D materials *in vitro* has primarily focused on interactions at the material-bacterial interface, including physical membrane perturbation and oxidative stress. Physical perturbation may result from edge-mediated membrane damage or sheet-mediated wrapping, whereas oxidative stress may involve ROS-dependent pathways or ROS-independent oxidation potentially mediated by electron transfer. These processes can compromise membrane integrity, resulting in cytoplasmic leakage and membrane depolarization, and may ultimately disrupt bacterial metabolic activity (Zou et al., 2016; Mei et al., 2020; Xie et al., 2023). Given the extensive body of *in vitro* antibacterial research on 2D materials (summarized in Table 1), we next examine how specific material properties shape the interface between 2D materials and bacterial cells and thereby modulate direct bacterial responses in simplified systems.

### 3.1 Nanosheet dimensions

Nanosheet dimensions, particularly lateral size and layer number, can influence how 2D materials interact with bacterial cells. Of the two parameters, lateral size has received the most attention in antibacterial studies, as it directly governs the contact area and edge density available for bacterial interaction. Systematic tuning of the lateral size of GO nanosheets revealed that GO-0, with the largest average lateral area of 0.753 μm^2^, exhibited stronger antibacterial activity than GO-240, with the smallest average lateral area of 0.010 μm^2^ (Liu et al., 2012). AFM analysis suggested that GO-0 more readily covered bacterial surfaces and occupied membrane active sites, thereby inhibiting bacterial proliferation. Another study showed that the size-dependent antibacterial behavior of GO was also influenced by the exposure conditions (Perreault et al., 2015). The authors found that large GO sheets mainly inhibited bacterial growth through cell wrapping in suspension, whereas on surfaces, smaller GO sheets induced stronger oxidative stress due to their higher defect density, resulting in stronger bactericidal activity.

In contrast to the finding that larger GO sheets showed stronger antibacterial activity in suspension assays, studies on other 2D materials have reported the opposite size-dependent trend. Zhao et al. separated MoS_2_ nanosheets with different lateral sizes and observed that antibacterial activity increased as the sheet size decreased (Zhao et al., 2021). Similarly, smaller Ti_3_C_2_T_x_ nanosheets with higher edge density can damage bacterial membranes and induce DNA leakage, leading to stronger bactericidal activity (Arabi Shamsabadi et al., 2018). Interestingly, a parabolic relationship between GO sheet size and antibacterial activity has been observed in the oral bacterium *S. mutans*, with intermediate-sized sheets showing the strongest effect (Yu et al., 2020). The authors proposed that different mechanisms, including membrane cutting and cell wrapping, may operate simultaneously across different size ranges.

One factor that may help explain these divergent trends is material-specific dispersion behavior. Owing to its abundant oxygen-containing functional groups, GO can remain relatively well dispersed under suitable aqueous conditions (Fadeel et al., 2018; Wang et al., 2024), allowing large sheets to retain sufficient accessibility for extensive surface coverage or wrapping. In contrast, some emerging 2D materials are more prone to restacking or aggregation (Ma et al., 2018; Li et al., 2022), which reduces the accessible sheet surface and limits bacterial contact. Under well-dispersed conditions, smaller nanosheets may retain a higher edge density and show stronger antibacterial activity.

In addition to lateral size, layer number may also influence antibacterial activity. In Langmuir-Blodgett deposited GO films on polyethylene terephthalate, antibacterial activity increased from one to three flat GO layers, reaching 89% inactivation of *E. coli* in the three-layer film. This increase coincided with greater GO coverage and material density, while the retained activity of the immobilized films indicates that edge-mediated cutting is not the sole mechanism underlying antibacterial activity (Mangadlao et al., 2015). Similarly, in GO coatings deposited on titanium, increasing layer number was accompanied by thicker, rougher, and more wrinkled coatings, together with enhanced antibacterial activity and higher bacterial ROS levels. These observations suggest that ROS-related oxidative stress may be a primary contributor to the antibacterial response, while “nanoknife” cutting and cell wrapping are limited by the immobilized configuration (Qiu et al., 2017). In prGO nanosheet dispersions tested by agar diffusion, the preparation with the lowest reported thickness (6.18 nm) showed the largest inhibition zones against four clinical isolates, whereas activity declined after apparent restacking into thicker aggregates. Reduced stacking may expose more material surface for bacterial contact and thereby enhance antibacterial activity in dispersed nanosheets (Baqi et al., 2026).

Critically, layer number does not appear to follow a universal relationship with antibacterial activity. In immobilized coatings, additional deposited layers increase material coverage and reshape the surface available for bacterial interaction. In dispersed nanosheets, reduced stacking can expose more of the material surface. Antibacterial activity is therefore more likely to depend on the effective contact interface between the material and bacterial cells than on layer number alone.

### 3.2 Surface chemistry

In addition to nanosheet dimensions, the surface chemistry of 2D materials also plays an important role in determining their antibacterial behavior, mainly by regulating the effective contact between nanosheets and bacterial cells. This contact can be influenced by surface functional groups, dispersion state, and electrostatic interactions with bacterial membranes.

For graphene-based materials, oxidation degree is closely related to the abundance of oxygen-containing functional groups, which can influence hydrophilicity, dispersion behavior, and membrane interaction. A systematic comparison of Gt, GtO, GO, and rGO demonstrated that the antibacterial behavior of GBMs is closely related to their surface chemical characteristics (Liu et al., 2011). A similar pattern has been observed for GDY, whose oxidized derivative showed enhanced antibacterial activity owing to the introduction of oxygen-containing functional groups that improved hydrophilicity, colloidal stability, and contact with bacterial cells (Zhang et al., 2019).

Such dispersion-related effects are relevant because aggregation or restacking can reduce the accessible nanosheet surface, limit the contact between materials and bacterial cells and thereby impair antibacterial processes. For example, single-layer MoS_2_ nanosheets showed significant antibacterial activity when well dispersed, whereas their antibacterial performance decreased markedly once they restacked and formed aggregates in solution (Yang et al., 2014). More recently, immobilizing MoS_2_ nanosheets within porous polymer beads was shown to prevent nanosheet aggregation and sustain antibacterial activity (Park et al., 2025). These observations suggest that dispersion state can influence the availability of active nanosheet surfaces and thereby affect the apparent antibacterial behavior of 2D materials.

Surface charge is another important chemical property influencing the antibacterial activity of 2D materials, as most bacterial cell membranes possess a net negative surface charge (Vejzovic et al., 2022). Therefore, electrostatic interactions between nanosheets and bacterial membranes can strongly influence antibacterial performance. Engineering the surface charge of Ti_3_C_2_ has been shown to significantly alter its biological activity. Modification of Ti_3_C_2_ with positively charged molecules can reverse its surface charge and enhance interactions with bacterial membranes, resulting in improved antibacterial activity (Rozmysłowska-Wojciechowska et al., 2020). Mixed-charge functionalization of MoS_2_ nanosheets produced tunable and Gram-selective antibacterial activity against MRSA and *P. aeruginosa*. Specifically, the neutral mixed-charge formulation 6P4N (containing 60% positive and 40% negative ligands) preferentially inhibited the Gram-positive strain MRSA, while the more positively charged formulation 8P2N (containing 80% positive and 20% negative ligands) showed stronger inhibition against the Gram-negative bacterium *P. aeruginosa* (Sahoo and De, 2022).

### 3.3 Material transformation in exposure media

Antibacterial activity is often interpreted from properties measured before exposure, although 2D materials can undergo significant transformation in aqueous media. For example, Ti_3_C_2_T_x_ oxidized in air, liquid, and solid environments, with the most rapid oxidation occurring in liquid media. In aqueous dispersions, TiO_2_ formation and marked conductivity loss occurred despite retained dark color and colloidal stability, indicating that apparent dispersion stability does not ensure preservation of the initial material state (Habib et al., 2019). Such changes may modify the material-bacterial interface and thereby alter antibacterial responses. Consistent with this possibility, Ti_3_C_2_T_x_ membranes aged in air for over 30 days showed more than 99% growth inhibition of both *E. coli* and *B. subtilis*, compared with 67% and 73%, respectively, for fresh membranes. The enhanced antibacterial activity was attributed to the oxidized TiO_2_/C surface layer formed during aging (Rasool et al., 2017).

Beyond surface oxidation, some 2D materials can undergo oxidative dissolution in aqueous media, producing soluble metal species whose contribution to antibacterial activity should be distinguished from that of the parent nanosheets. In a vertically aligned MoS_2_ coating system, released molybdate (MoO_4_^2-^) was proposed to contribute to an inhibition zone against the Gram-positive bacterium *S. aureus*, whereas no inhibition zone was detected against the Gram-negative bacterium *E. coli* (Tang et al., 2020). In a later study, MoS_2_ nanosheets, but not sodium molybdate, inhibited *E. coli* in nitrate-containing medium. This difference was attributed to an activity at the MoS_2_ surface resembling that of nitrite reductase, which converted nitrite into antibacterial nitric oxide (NO). Sodium molybdate did not support this reaction (Peng et al., 2023a).

Together, once transformation occurs, antibacterial activity can no longer be inferred from the parent nanosheets alone. Comparisons among the original material, transformation products, and soluble species are therefore needed to resolve their individual contributions.

### 3.4 Bridging *in vitro* findings to *in vivo* complexity

Taken together, these studies show that the intrinsic antibacterial activity of 2D materials may partly contribute to the microbial depletion observed in some *in vivo* studies. However, translating such findings to gut microbial ecosystems remains challenging. Most *in vitro* antibacterial assays rely on standard model strains such as *E. coli* and *S. aureus*, which primarily serve as Gram-negative and Gram-positive models rather than representative members of the gut microbiota. Moreover, the majority of studies have used single bacterial strains to investigate the direct antibacterial effects of 2D materials while neglecting interspecies interactions. For example, *Vibrio* employs type VI secretion systems to exclude competitors like *Aeromonas*; thus, a decrease in *Aeromonas* may not be due to the direct effects of material exposure but rather to the proliferation of *Vibrio* (Logan et al., 2018). Therefore, *in vitro* assays based on a single or limited number of model strains cannot fully reflect how 2D materials influence complex gut microbial communities *in vivo*.

## 4 Future perspectives

Current studies on the effects of 2D materials on the gut microbiota have primarily focused on *in vivo* microbiota alterations, complemented by *in vitro* mechanistic investigations on the antibacterial effects. While these studies have provided valuable insights, several knowledge gaps remain. To support a more integrated understanding of how 2D materials interact with the gut microbiota and to inform the design of safer 2D materials, several directions for future research are outlined below.

First, future studies should extend investigations of gut microbiota responses beyond GBMs to emerging 2D material classes. Given their distinct elemental compositions, surface chemistries, and transformation behaviors in the gastrointestinal environment, response patterns established for GBMs may not be directly transferable to other materials. Comparative *in vivo* studies conducted under harmonized characterization and exposure frameworks would help identify effects specific to individual material classes and broaden the current evidence base.

Second, linking material properties to microbiota responses and subsequent host effects *in vivo* remains a central challenge. *In vitro* studies have shown that physicochemical properties of 2D materials such as lateral size, oxidation state, and surface charge can influence bacterial behavior. However, whether these effects are recapitulated in the intestinal environment remains unclear. Current *in vivo* studies largely describe shifts in microbial composition without directly relating these changes to material characteristics. To establish reliable structure-activity relationships, future studies can first generate dose-response curves under comparable exposure conditions, rather than interpreting material effects from single-dose observations. In parallel, a basic characterization framework should be adopted across studies, including lateral size distribution, thickness or layer number, surface chemistry, zeta potential, and hydrodynamic diameter in relevant exposure media. Such standardized dose reporting and material characterization would improve comparisons across studies and help determine whether microbiota alterations follow predictable material-dependent patterns.

Third, greater attention should be paid to the dynamic nature of microbiota responses. The gut microbiota is not a static system, yet most existing studies rely on single endpoint measurements. Emerging time-series analyses indicate that microbiota responses to nanomaterial exposure can evolve rapidly, with distinct stage-specific patterns that are not captured by endpoint-based designs (Lin et al., 2025). Capturing these temporal trajectories will be critical for distinguishing transient perturbations from sustained dysbiosis and for understanding how microbiota changes relate to subsequent host responses.

Fourth, *in vitro* models need to better reflect the ecological complexity of microbial communities. Most current *in vitro* studies rely on single-strain assays, which are insufficient to explain the community-level alterations observed *in vivo*. Microbial functions are shaped by interspecies interactions, and even low-abundance taxa can exert disproportionate effects on community behavior (Rolig et al., 2015). Advances, including microbiota transplantation and *in vitro* systems incorporating whole microbial communities, provide more realistic platforms by preserving community structure and metabolic interactions (Rawls et al., 2006; Vrancken et al., 2019). Such approaches will be essential for determining whether microbiota alterations observed *in vivo* arise from direct interactions between nanomaterials and microbial communities, rather than indirect effects mediated by the host.

Finally, the bidirectional interactions between microbiota and nanomaterials, particularly microbial transformation processes, warrant further investigation. The gut microbiota should not be viewed solely as a passive target of exposure. Increasing evidence suggests that microbial communities can actively transform nanomaterials within the intestinal environment. Engineered inorganic carbon nanomaterials (single-walled carbon nanotubes and GO) have been shown to be converted into short-chain fatty acids through microbial fermentation, with carbon from nanomaterials entering microbial metabolic pathways (Cui et al., 2023). Understanding these transformation processes will be critical for interpreting exposure outcomes and for assessing the long-term biological fate of nanomaterials (Lv et al., 2025).

Taken together, these gaps indicate that future research should not only clarify the mechanistic interactions between 2D materials and the gut microbiota, but also consider their long-term biological implications under realistic exposure scenarios. More consistent material characterization and microbiota-relevant testing models will help improve cross-study comparability and provide a more practical basis for risk evaluation and safer design of emerging 2D materials.

## 5 Conclusion

Overall, current evidence suggests that gut microbiota dysbiosis is an important biological response to 2D material exposure. *In vivo* studies have documented microbial alterations and associated host effects, whereas *in vitro* studies have revealed property-dependent antibacterial activity. However, existing *in vitro* models remain too simplistic to fully recapitulate the complexity of microbial responses observed *in vivo*. Future progress will depend on improved structure-activity relationship analysis *in vivo*, temporal investigations of microbiota dynamics, advanced *in vitro* microbial community models, and a deeper understanding of material biotransformation by the gut microbiota. Such efforts will strengthen the safety assessment of 2D materials and advance our understanding of their interactions with the gut ecosystem.
